# Vaginal Endosalpingiosis Case Report: A Rare Entity Presenting as Intermenstrual Bleeding

**DOI:** 10.1155/2017/2424392

**Published:** 2017-11-09

**Authors:** Sara Câmara, Gustavo Mendinhos, Rosa Madureira, Amália Martins, Carlos Veríssimo

**Affiliations:** ^1^Department of Gynecology, Hospital Dr. Nélio Mendonça, Av. Luís de Camões, Funchal, 9004-514 Madeira Island, Portugal; ^2^Department of Gynecology, Hospital Beatriz Ângelo, Avenida Carlos Teixeira 514, 2674 Loures, Portugal

## Abstract

Endosalpingiosis is a benign and rare entity whose pathophysiology remains unknown. It has been described in pelvic organs, the abdomen, or axillar lymph nodes. Its underrecognition has occasionally led to its misinterpretation for an adenocarcinoma. This case reports the treatment and follow-up of vaginal endosalpingiosis, presenting as a vaginal polyp in a premenopausal women with intermenstrual bleeding. To our knowledge this is the first reported case of vaginal endosalpingiosis and the second mucosal localization after bladder endosalpingiosis.

## 1. Introduction

Endosalpingiosis is a benign condition defined by the presence of ectopic fallopian tube-type epithelium. Histologically it corresponds to ciliated tubal-like epithelium lining glandular cysts [1] (Figure 2).

The Mullerianosis theory defends that endosalpingiosis arises from embryonic Müllerian tissue, misplaced during organogenesis. According to this theory adenomyosis, endometriosis, and endocervicosis, which frequently coexist [2], originate in embryonic remnants which share the same origin and are curable by healthy margins surgical excision [3]. Other authors defend a metaplastic origin in the peritoneal mesothelium [4, 5] and still others defend an implantation process where these cells migrate and proliferate in the harbouring organ [6].

Endosalpingiosis is influenced by the hormonal status and is most often identified in premenopausal women (similar to endometriosis). However it has also been described in postmenopausal elderly women [5] and in men [4].

Intraoperatively, endosalpingiosis has often been taken by endometriosis, even if they frequently coexist [1]. Fulguration of lesions without biopsy can contribute to endosalpingiosis underrecognition [1]. Endosalpingiosis is usually localized in the pelvis (ovaries, fallopian tube, uterine serosa and myometrium [7], bladder [5, 6], and pelvic peritoneum [8]). Outside the pelvis it has been identified in the retroperitoneal [9] or axillary [10] lymph nodes (presenting as Müllerian type glandular inclusions), in the omentum, in the bowel serosa, and in the umbilicus [11].

The finding of this pathological diagnosis is usually incidental [12], though endosalpingiosis has been associated with pelvic pain [12, 13], menorrhagia [1, 7], hematuria [5], or a symptomatic mass in the pelvis [7].

Although endosalpingiosis is a benign condition, it can invade the* lamina propria* and the* muscularis mucosa* and therefore be misinterpreted for well differentiated, invasive adenocarcinoma [5, 6]. When it appears in lymph nodes, for instance, it can resemble metastatic carcinoma [10]; biopsies from diffuse peritoneal protrusions can mimic malignancy [8]; and peritoneal washings can be interpreted as a positive peritoneal cytology for malignancy [14]. The misdiagnosis of endosalpingiosis is even more likely if it appears in men, due to the rarity of this condition in that population [4]. The differential diagnosis of endosalpingiosis is of great clinical relevance and some histological characteristics have been described which could help in this challenge (the presence of ciliated cells, immunohistochemical findings, others) [1, 10].

While endometriosis frequently affects the vaginal cuff and endocervicosis has previously been recognized in the vagina [15], to our knowledge this is the first published case of vaginal endosalpingiosis.

## 2. Case Presentation

A 46-year-old woman presented with intermenstrual bleeding since several months before. She had 2 previous cesareans and no other abdominal or pelvic surgery. With no active sexual life, this patient had not been taking hormonal contraception for years and she used to have regular menses with normal bleeding before the present situation.

This woman had no history of infertility (with 2 pregnancies at 19 and 21 years old) and denied present or past symptoms of dysmenorrhea or dyspareunia.

**Figure 1:**
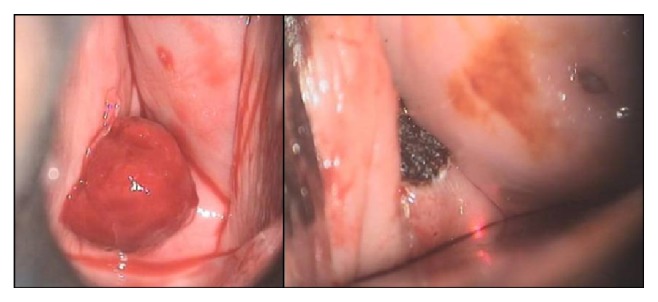
Vaginal polyp before and immediately after excision with electrocoagulation of the polyp base (both photos have been obtained and shared with the patient's informed consent).

Her clinical evaluation revealed a polypoid neoplasm, in the posterior vaginal cuff, about 2 cm. It had a soft consistence and was bleeding easily with touch. The uterine cervix or vaginal walls were otherwise completely normal to macroscopy (Figure 1).

Her cervical smear was negative for intraepithelial lesion or malignancy, and the gynecological ultrasound revealed only small uterine myomas, with normal adnexa, and no signs of endometriosis.

This patient was electively admitted for ambulatory polypectomy with electrocoagulation of the bleeding insertion pedicle (Figure 1).

**Figure 2:**
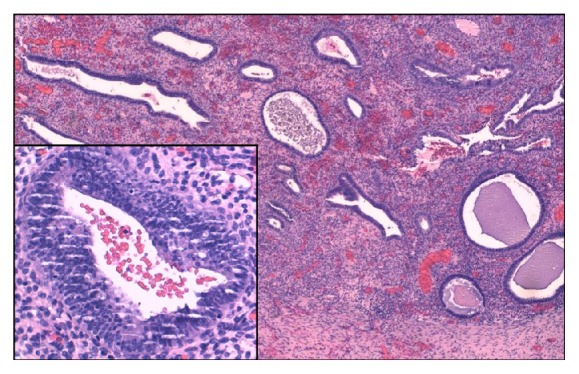
Multiple cysts lined by ciliated tubal-like cuboidal epithelial cells without atypia (Hematoxylin and Eosin ×4. Inset: Hematoxylin and Eosin ×20).

**Figure 3:**
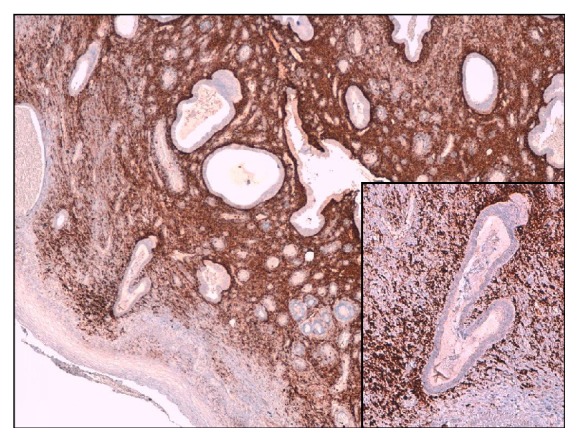
Blunt papillary projections are evidenced by CD10 antigen, expressed in stromal cells (CD10 immunohistochemistry ×4. Inset: CD10 ×10).

Pathology examination identified a stratified squamous epithelium with submucosal endosalpingiosis and signs of recent bleeding (Figures 2 and 3).

At subsequent follow-ups she was asymptomatic and the vaginal cuff cicatrized easily with no sequel. After 1 completed year, the patient remains asymptomatic and the vagina is macroscopically unremarkable.

## 3. Discussion

Endosalpingiosis' origin, risk factors, prognosis, and follow-up recommendations remain unestablished. In the presented case either the embryonic remnant theory, the implantation theory, or the metaplastic theory is valid hypothesis. The only relevant fact in our patient personal history was the 2 previous cesareans; however, the longtime interval between this surgical insult and the diagnosis of endosalpingiosis is against the establishment of a causal relation.

Intermenstrual bleeding was the only symptom of our patient. Even without hormonal contraception, she had no complaint of dysmenorrhea, for instance. In fact this lesion could have gone undetected if it would have been smaller, in an inattentive observation.

Considering the appearance of this easy bleeding polypoid lesion, we opted for elective complete polypectomy with subsequent electrocoagulation, avoiding a potential bleeding complication. Also, the polypectomy specimen was more representative than a biopsy sample for pathological evaluation, avoiding misdiagnosis. On the other hand, a surgical excision with healthy margins could have represented an overtreatment, with more probabilities of a defective scar, or other comorbidities.

As mentioned above, to the authors' best knowledge, this is the first reported case of vaginal endosalpingiosis. While representing a diagnostic challenge for the pathologist, maybe the most important concerns for the surgeon would be to provide enough and undeteriorated specimen for a correct pathological diagnosis and avoid overtreatment.
